# A randomized controlled trial of different methods of alcohol screening and brief intervention in routine probation settings: 12-month outcomes

**DOI:** 10.1186/1940-0640-7-S1-A82

**Published:** 2012-10-09

**Authors:** Dorothy Newbury-Birch, Eileen Kaner, Paolo Deluca, Simon Coulton

**Affiliations:** 1 Institute of Health and Society, Newcastle University, Newcastle upon Tyne, UK; 2 Institute of Psychiatry, King's College London, London, UK; 3 Center for Health Service Studies, University of Kent, Canterbury, UK

## 

A large number of randomized controlled trials in health settings have consistently reported positive effects of BI to reduce risky alcohol use. However, although alcohol misuse is common amongst offenders, there is limited evidence of alcohol BI in the criminal justice system. The Screening and Intervention Program for Sensible Drinking (SIPS) Criminal Justice System trial (SIPS-CJS), a prospective pragmatic cluster randomized control trial, was the first large multicenter trial of alcohol screening and BI in the CJS carried out in England. Offender managers (n = 227) from 20 probation offices were randomized to one of three conditions: patient intervention leaflet only [PIL], brief advice [BA], or brief lifestyle counseling [BLC]) and to one of two screening tools (the Modified Single Alcohol Screening Question [M-SASQ] or the Fast Alcohol Screening Test [FAST]). The primary hypothesis was that BLC delivered by an alcohol health worker would be more effective than BA or PIL delivered by CJS staff. Outcomes were assessed at six and 12 months. The mean age of participants was 31, and the mean Alcohol Use Disorders Identification Test (AUDIT) score at baseline was 16.1. The majority of the sample were male (85%), white (76%), and current smokers (79%). Sixty-seven percent of participants were available for follow-up at six months post-intervention, and 59% were available at 12 months. No significant differences were found in follow-up rates between the intervention groups. At 12-month follow-up, ratings were high in all groups in terms of general satisfaction, communication, and interpersonal manner. At 12 months, the proportion of participants positive for AUDIT overall had been reduced by 15.6%. This reflected a decrease of 18.6% in the PIL group, 14.4% in the BA group, and 13.7% in the BLC group. An adjusted logistic regression model found no significant effects of intervention group, screening approach, or baseline AUDIT score.

